# Acquired Partial Lipodystrophy: Clinical Management in a Pregnant Patient

**DOI:** 10.1210/jendso/bvae181

**Published:** 2024-10-21

**Authors:** Martina Romanisio, Leonardo Bighetti, Tommaso Daffara, Edoardo Luigi Maria Mollero, Caterina Pelosini, Valentina Antoniotti, Carola Ciamparini, Gianluca Aimaretti, Marina Caputo, Flavia Prodam

**Affiliations:** Endocrinology, Department of Translational Medicine, Università del Piemonte Orientale, Novara 28100, Italy; Endocrinology, Department of Translational Medicine, Università del Piemonte Orientale, Novara 28100, Italy; Endocrinology, Department of Translational Medicine, Università del Piemonte Orientale, Novara 28100, Italy; Endocrinology, Department of Translational Medicine, Università del Piemonte Orientale, Novara 28100, Italy; Chemistry and Endocrinology Laboratory, Department of Laboratory Medicine, University Hospital of Pisa, Pisa 56126, Italy; Endocrinology, Department of Translational Medicine, Università del Piemonte Orientale, Novara 28100, Italy; Department of Health Sciences, Università del Piemonte Orientale, Novara 28100, Italy; Endocrinology, Department of Translational Medicine, Università del Piemonte Orientale, Novara 28100, Italy; Endocrinology, Department of Translational Medicine, Università del Piemonte Orientale, Novara 28100, Italy; Endocrinology, Department of Translational Medicine, Università del Piemonte Orientale, Novara 28100, Italy; Department of Health Sciences, Università del Piemonte Orientale, Novara 28100, Italy; Endocrinology, Department of Translational Medicine, Università del Piemonte Orientale, Novara 28100, Italy; Department of Health Sciences, Università del Piemonte Orientale, Novara 28100, Italy

**Keywords:** acquired partial lipodystrophy, pregnancy, leptin levels, glucose levels, triglycerides

## Abstract

**Background:**

Pregnancy represents an additional challenge to the complex clinical picture of lipodystrophy disorders, and the management of such conditions with related comorbidities has been underreported. This work aims to outline the risk associated with a pregnancy event for women dealing with acquired partial lipodystrophy and the need for diverse but specialized care.

**Case:**

We report on the successful pregnancy outcome of a 28-year-old woman with an acquired partial form of lipodystrophy related to an allogenic bone marrow transplant that occurred at pediatric age. Although metabolic control was challenging, glucose levels progressively improved during the pregnancy, and triglycerides increased less than expected. The periodic monitoring of leptin levels showed a progressive increase with a peak in the third trimester (41.53 ng/mL), followed by a fast decline the day after giving birth, with a lower basal level than the prepregnancy period. However, preterm delivery occurred associated with cardiac complications in the mother.

**Results:**

A total of 12 studies were retrieved concerning women aged 14 to 38 years with various lipodystrophy phenotypes. Diabetes and hypertriglyceridemia were the most common comorbidities. Most women had successful pregnancies despite gestational complications (including miscarriages), preterm and emergency deliveries, and newborns undergoing partum or postpartum transient or chronic complications.

**Conclusion:**

Lipodystrophy disorders expose both mothers and children to very high risk. Intensive monitoring and care of all potential clinical complications should be planned and carried out by a multidisciplinary team before, during, and after the pregnancy. Leptin secretion during pregnancy should be investigated more deeply in these patients.

Lipodystrophy syndromes are rare and heterogeneous, characterized by a loss (or deficiency) of subcutaneous adipose tissue, abnormal fat deposition in ectopic areas, and decreased leptin levels. Metabolic impairment, insulin resistance, hypertriglyceridemia, hepatic steatosis, diabetes, and dyslipidemia are typical comorbidities observed within this syndrome [1, 2]. Physical examinations and clinical history allow the assessment of distinct body composition (with laboratory findings) and glucose/lipid metabolic impairment [3], leading to a conclusive diagnosis of lipodystrophy. However, due to the low prevalence and heterogeneity, lipodystrophy may frequently be unrecognized or misdiagnosed among other diseases [1, 4].

Effective management of lipodystrophy includes lifestyle changes, such as diet and exercise. Plastic surgery and psychological support can improve the well-being of some patients [1], as well as evidence-based medical treatment of specific metabolic comorbidities, to manage the short- and long-term complications of the disease. The need to treat metabolic comorbidities may be reduced with leptin replacement therapy [5, 6].

Lipodystrophy disorder is classified as genetic or acquired, partial or generalized. In the context of acquired partial lipodystrophy (APL), bone marrow transplantation (BMT), especially in conjunction with total body irradiation, is considered a high-risk factor for the development of the disease [7-16].

Given the complexity and rarity of the disease, with an estimated global prevalence of 1.3 to 4.7 cases/million [17], pregnancy represents a delicate and challenging event, and the management of this condition is underreported in the literature (in the 2 two decades, of around 1300 studies reporting on lipodystrophy cases, 10-15 studies concerned its management during a pregnancy). To provide additional knowledge on this topic, this paper aims to provide an overview of the complexity of lipodystrophy disorder during pregnancy, its challenging management, and the associated increased risk of pregnancy complications. The first case report in an APL secondary to BMT is also presented to provide a practical example of clinical management.

## Case Report

We report a case of a pregnant 28-year-old Caucasian female followed for diabetes and hypertriglyceridemia since she was 21 years old in the context of APL. Past medical history included childhood lymphocytic leukemia at 8 years old, treated with radiotherapy and chemotherapy (CT) (AIEOP-BFM LLA 2000 protocol—prephase: prednisone IV + methotrexate (MTX) intrathecal (IT)—induction: vincristine IV + prednisone IV + daunorubicin IV + asparaginase intramuscular + MTX IT—consolidation: cyclophosphamide IV + mercaptopurine oral + cytarabine IV + MTX IT), and allogeneic BMT. At the age of 13 years, she developed CT-related heart disease with left ventricular dysfunction [ejection fraction (EF) 45%] that was treated temporarily with β-blocker and angiotensin-converting enzyme inhibitors; it normalized during follow-up.

At the first physical examination in our center, the 26-year-old patient reported a cushingoid face, buffalo hump, loss of subcutaneous adipose tissue in the lower and upper limbs with accumulation in the suprascapular area, acanthosis nigricans, and defluvium capitis. She was of normal weight (weight 46 kg, body mass index: 21 kg/m^2^). She described that Cushing features were slightly present in infancy during the acute phase of cancer treatment. However, changes in her body composition and face started becoming more apparent some years later, mainly after puberty.

Excluding autoimmune conditions, diabetes was reclassified from type 1 to a lipodystrophy-related type. C-peptide was maintained (1.32 ng/mL at fasting). Insulin resistance was confirmed by the daily insulin amount (3.13 UI/Kg/24 hours) and the insulin dose-adjusted hemoglobin A1c (HbA1c) index (23.7 ng/mL) [18]. Cushing syndrome was excluded [normal cortisol inhibition after suppression test with dexamethasone 1 mg (0.5 µg/dL) and normal urinary free cortisol (4.4 µg/24 hours)]. Androgens (17 OH progesterone: 0.51 ng/mL, testosterone: 12.2 ng/mL, DHEAS: 121.5 µg/dL) and thyroid function (TSH: 2.763 µUI/mL, fT4: 1.18 ng/dL) were normal.

Initial treatment concerned insulin (1.06 UI/kg, glargine total dose 14 UI/day, lispro total dose 36 UI/day), quickly titrated to high doses (3.13 UI/kg, glargine total dose 52 UI/day, lispro total dose 95 UI/day) because diabetes was not adequately controlled at ambulatory glucose profile [AGP; time in the range (TIR) 5%, time above range (TAR) 95%, time below range (TBR) 0%, glucose management indicator (GMI) 11.3%]; metformin (1 g/day) and semaglutide (0.25 mg/week) were added. Nocturnal hyperphagia partially improved by semaglutide treatment. An increased dose of semaglutide was not well tolerated due to nausea. Furthermore, the patient refused the use of a continuous subcutaneous insulin infusion. Hypertriglyceridemia was discreetly controlled (182 mg/dL) with polyunsaturated fatty acid (3 g/day) and fenofibrate (145 mg/day). With a long clinical history of amenorrhea (FSH: 14 mUI/mL, LH: 6 mUI/mL, estradiol < 30 pg/mL, multifollicular ovaries at ultrasound at baseline), she had her first period after changing the treatment, without introducing progesterone or estrogens.

A short time later, at the age of 27 years, she became unexpectedly pregnant. Therefore, semaglutide, metformin, fenofibrate, and angiotensin-converting enzyme inhibitors were stopped; polyunsaturated fatty acid was reduced; and acetylsalicylic acid was added after gynecological consultation.

During the first trimester of pregnancy, AGP reports improved (TIR 27%, TAR 73%, TBR 0%, GMI 8.5%), whereas triglycerides (TGs) increased but less than expected during pregnancy (548 mg/day).

In the second trimester, AGP reports kept improving [TIR 55%, TAR 44% (30 + 14), TBR 1% (1 + 0), GMI 7.6%] after increasing the insulin dose and resuming metformin, as well as the hypertriglyceridemia state (296 mg/dL). As TSH values were inadequate for gestational age (3.44 UI/mL), levothyroxine was started as a precaution. At 16 weeks of gestation, cervical cerclage was performed, and progesterone therapy started.

During the third trimester, further improvement in glycemic control (TIR 77%, TAR 19%, TBR 4%, GMI 6.5%) was observed, with a TIR of 77%. In the third trimester, she had a total weight gain of 9.5 kg.

Furthermore, during pregnancy, the patient developed severe periodontitis with progressive total tooth loss, needing surgery procedures to decrease the widespread infection risk.

Despite achieving good glycemic control, at 33 weeks of gestation, the patient developed acute pulmonary edema followed by an emergency cesarean delivery.

The echocardiographic evaluation showed severe left ventricular diastolic dysfunction (EF 30%), treated with invasive ventilation, diuretics, nitroglycerin, inotropic drugs, and ivabradine. A cardiac magnetic resonance imaging showed a left ventricular dilatation and depression of systolic function without alterations in tissue characterization or adipose tissue depots, a probable consequence of previous treatment with anthracyclines.

Furthermore, suspecting the development of Sheehan syndrome due to hyponatremia and hypoprolactinemia, after delivery, the patient started cortisone acetate as a precautionary measure, which was interrupted due to evident normal hypothalamic-pituitary-adrenal function several weeks later.

**Figure 1.**
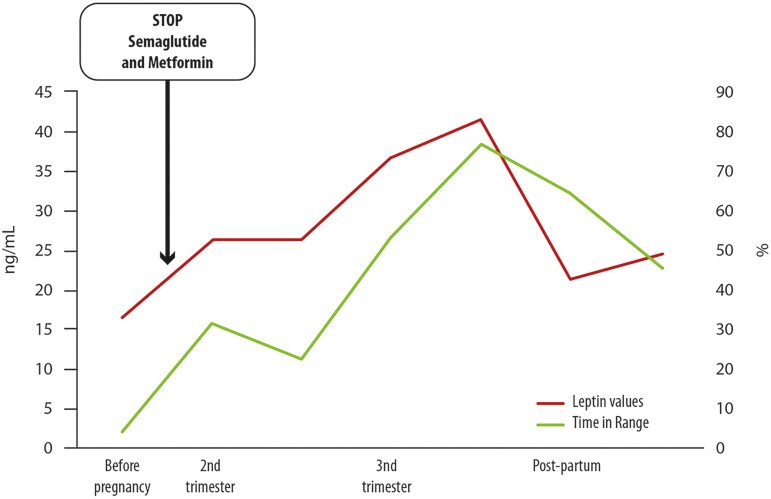
Trend of leptin values (ng/mL) and glucose time in range (percent) at flash glucose monitoring during and soon after pregnancy.

Since glucose levels improved during pregnancy and TGs increased less than expected, leptin values were periodically monitored during pregnancy and in the early postpartum period: increased levels were progressively observed during pregnancy with a peak in the third trimester (41.53 ng/mL), followed by a fast decline the day after giving birth, with lower basal level than the prepregnancy period (Fig. 1). The newborn was in good general condition (appropriate for gestational age, 2136 g) except for the development of mild hypoglycemia after birth and the finding of triple X syndrome. Leptin levels in the baby were 7.05 ng/mL.

Given the recent clinical history of heart failure, the previous therapy was restarted after childbirth; an SGLT2 inhibitor was included together with a temporary heart device, which was removed upon recovery of ventricular function.

In the 2 years after pregnancy, diabetes progressively deteriorated with a variable HbA1c (7.3-8.6%) during diet, insulin treatment (3 UI/kg/die), SGLT2 inhibitor, and semaglutide (0.25 mg/weekly). TGs were partially controlled (290-540 mg/dL) during treatment with fenofibrate, omega 3 (4 g/die), and MCT oil (15 mL/die). She returned to the basal weight.

## Literature Review

Papers were identified by conducting a PubMed search using the following keywords or combinations: lipodystrophy AND pregnancy; lipoatrophy AND pregnancy. Only studies on humans published in English in the last 25 years were considered. The articles were screened (full-text or abstract only) according to their relevance to the topic, as judged by the authors. Specific information deemed relevant for the analysis was extracted for each study. In particular, the analysis focused on data related to the age of the patient, the type of lipodystrophy, clinical history of the patient, clinical features at physical examination, conditions developed or influenced by the pregnancy (including leptin values if reported), pregnancy outcome, and finally clinical state after childbirth.

## Results

Twelve studies were selected and investigated further for the purpose of this work. The relevant information has been summarized in Table 1.

**Table 1.** Overview of studies reporting on pregnancy management and outcome in patients affected by lipodystrophy

Reference	Number of patients and age of pregnancy	Lipodystrophy type, clinical history, and additional features	Clinical development during pregnancy	Delivery, pregnancy outcome, and newborn conditions	Postpartum clinical conditions of the patient	Leptin values trend across the pregnancy	Treatments applied across the pregnancy
Castell et al 2012 [19]	23	FPLD3 (*PPARγ* mutation) Hepatic steatosis, hypertension, diabetes (HbA1c of 8%), hypertriglyceridemia (plasma TG at 2.55 mmol/L), hirsutism	Diabetes controlled (HbA1c < 6%), no hypertension or proteinuria	Premature delivery at 31 weeks of a child who developed hydrops fetalis and died 24 hours later	NR	Low level of plasma leptin 4.7 ng/mL (early-stage pregnancy). Further changes not reported	High daily insulin doses (200 IU) until delivery
Dolberg et al 2000 [20]	23	General acquired lipodystrophy. Diabetes (plasma glucose maintained within 300 mg/dL), hypertriglyceridemia, acute pancreatitis, hirsutism, abdominal pain, constipation alternating with diarrhea, polydipsia and polyuria, upper GI hemorrhage, esophageal and gastric varices, and cirrhosis.Testosterone level of 13 ng/dL, 17α-hydroxyprogesterone of 83 ng/dL, estradiol level of <20 pg/mL, in conjunction with normal progesterone, cortisol, and thyroid functions	HbA1c values were consistently within 0.2% of the nondiabetic range. At 26 weeks of gestation, premature contractions and GI bleeding	Previous experience of an early first-trimester spontaneous abortion. Emergency C-section at 28 weeks. Newborn had mild clitoromegaly and mild hirsutism but no evidence of carbohydrate intolerance or leprechaunism	Hospitalization for hyperglycemia after delivery. Development of tinnitus in association with gradual and profound hearing loss; 3 months after childbirth, massive GI hemorrhage and death	NR	U-500 regular insulin, at an initial dosage of 250 U 4 times a day. Treated with a transdermal patch of conjugated equine estradiol (0.5 mg, transdermal therapeutic system) Later, cyclic progesterone therapy; then given a trial of hydrocortisone and then prednisone. Initiation of hyperalimentation necessitated an insulin drip as high as 600 U/hour
Panchal et al 2005 [21]	27	FPLD2 (Dunnigan type related to *LMNA* gene) Hypertension and no proteinuria	Pregnancy was uneventful until 17 weeks. Proteinuria at 26 weeks. Gestational diabetes at 28 weeks (fasting serum glucose 4.8 mmol/L, 2-hour serum glucose post 75 grams oral glucose challenge 8.4 mmol/L). Hypertension (142/98 mmHg) with increasing proteinuria (1.38 g/24 hours with a creatinine clearance of 82 mL/min) at 31 weeks	C-section at 39 weeks. The child was in good health	Complete resolution of her hypertension; proteinuria still present (0.8 grams/24 hours with a creatinine clearance of 56 mL/min), but normal renal function (urea 5.1 mmol/L, creatinine 84 mmol/L, sodium 139 mmol/L, potassium 3.9 mmol/L)	NR	Insulin from 29 weeks (novorapid 4 units each evening). Oral nifedipine SR 20 mg due to persistent hypertension. Insulin and antihypertensive medication were discontinued postpartum
Nijjar et al 2014 [22]	26 (first pregnancy) 31 (second) 38 (fourth)	FPLD2 (Dunnigan variety-related to *LMNA* gene) Poor glycemic control (postnatally diagnosed with T2D)	First uncomplicated pregnancy During second pregnancy, GD developed (2-hour reading on a 75 g GTT 11.5 mmol/L). During third pregnancy, poor glycemic control (HbA1C 8.8%) During fourth pregnancy, glycemic control deteriorated (HbA1C 7.7%). In the week preceding delivery, the HbA1C was 5.6% and TG level 3.1 mmol/L	First uncomplicated delivery. second delivery via emergency C-section at week 34 and preterm male delivered with cerebral ischemic injury third abortion in the first semester fourth delivery via C-section at 33 weeks. The infant required management of hypoglycemia	Glycemic control achieved	NR	During her second pregnancy, the maximum total daily insulin requirements just prior to delivery were 92 units. During her third pregnancy, 110 units daily. During her fourth pregnancy, over 250 units of insulin daily and maximal doses of metformin (2 g) from 12 weeks’ gestation. By 28 weeks’ gestation, IV insulin infusion with an average of 1 unit/hour and subcutaneous insulin therapy restarted after 2 days Later, glibenclamide 7.5 mg was prescribed in addition to 250 units of insulin and metformin 2 g daily. At 33 weeks’ gestation after administration of steroids for fetal lung maturation. Following delivery, pioglitazone and metformin for glycemic control
Maguire et al 2012 [23]	23	CGL1 (*AGPAT2* gene mutation) Hypertriglyceridemia (TG level 1355 mg/dL), insulin resistance, HbA1c level was 11.7%, serum insulin was 599 microunits/mL. After leptin treatment HbA1c level dropped to 7.4% and TG level fell dramatically. Just before conception, she had a normal HbA1c level of 5.7% and TG level of 75 mg/dL	Gain of 22 kg, increase in glycemic levels; Hb A1c level was 5.8% at 33 weeks of gestation	At 37 weeks, vaginal delivery was complicated by cephalopelvic disproportion with shoulder dystocia. The child had postpartum respiratory distress and Erb's palsy; otherwise, the child's growth was normal	Glycemic levels normalized, glucose levels were 70-110 mg/dL off of insulin	NR On leptin treatment	1200 units of insulin daily to maintain euglycemia. Experimental trial of recombinant leptin therapy, initially pediatric dose of 0.04 mg/kg/day, increased gradually to normalize her metabolic parameters. Metformin alone for glycemic control. During pregnancy, metformin stopped, while leptin continued at 0.13 mg/kg/day divided into 2 daily doses. During the latter half of pregnancy, up to 90 units of regular insulin 3 times daily. Leptin dose (0.13 mg/kg/day) without interruption after delivery
Madhra 2012 [24]	19	FPLD3 (*PPARγ* gene mutation). Hypertension, hypertriglyceridemia, diabetes	At 6 weeks’ gestation, severe hypertriglyceridemia (cholesterol 8.1 mmol⁄l and TG 20.1 mmol⁄l) and pancreatitis, deterioration of glycemic levels. (HbA1c of 140 mmol⁄mol, 15%). No increased hypertension. After treatment, HbA1c rapidly improved to 97 mmol⁄mol (11%). At 17 weeks’ gestation with acute pancreatitis secondary to hypertriglyceridemia (TGs > 100 mmol⁄l). During the next month, her HbA1c improved to 65 mmol⁄mol (8.1%). By 26 weeks’ gestation, her glycemic control had deteriorated	C-section at 32 weeks. The child had a developmental delay	Controlled glycemic levels by treatment	Normal levels before pregnancy	Twice-daily insulin converted to a basal-bolus regimen to improve glycemic control, folic acid 5 mg and aspirin 75 mg once daily and atorvastatin discontinued. At 17 weeks’ gestation, almost 300 units of IV insulin per day (5 units⁄kg) to maintain glycemic control. Then, a continuous subcutaneous insulin pump was used for the delivery of large doses of basal insulin. Mealtime boluses administered by subcutaneous injections using a carbohydrate-counting approach. By 26 weeks’ gestation, fenofibrate 200 mg and atorvastatin 40 mg at night were prescribed after her third plasma exchange. Antenatal betamethasone for fetal lung maturation required 20 units of insulin/hour to maintain glycemic control
Belo et al 2015 [25]	24	FPLD2 (Dunnigan variety-related to *LMNA* gene). Diabetes, dyslipidemia, acute pancreatitis	Loss of fat from the extremities and trunk and an excess of subcutaneous fat in the chin at week 7. Hypertriglyceridemia (TG levels of 1566 mg/dL). Controlled glycemic levels by treatment (HbA1c 4.7%). Acute pancreatitis at week 12 (TG levels of 9975 mg/dL). After fasting + fenofibrate treatment, TG levels 655 mg/dL. One week later, TG levels of 2965 mg/dL. After fasting-centered metabolic control, TG levels of 1086 mg/dL. After Omacor + Protifar treatment, TG levels remained constant between 1000 and 2000 mg/dL until the birth	Labor dystocia, C-section. No complications for the newborn	Suboptimal TG levels (TG levels of 882 mg/dL) and glycemic levels by treatment (HbA1c < 7%)	NR	At 7 weeks of pregnancy, the initial insulin total dose of 106 U/day (1.8 U/kg/day). Treated also with heparin and salmon oil. Statin, niacinic acid, and fenofibrate treatment are suspended when pregnancy is confirmed. Treatment with gemfibrozil 600 mg twice a day for hypertriglyceridemia. At 12 weeks’ gestation, fasting + fenofibrate 267 mg, once a day. Then, rigorous dietary plan and Omacor® and Protifar®. After delivery, returned to statin (rosuvastatin 20 mg), cholestyramine (4000 mg once a day), and fibrate (fenofibrate 267 mg once a day) treatment. Metformin was added to insulin
Morse et al 2000 [26]	29	Familial partial lipodystrophy (Köbberling-Dunnigan syndrome). Diabetes	Hypertriglyceridemia	Oxytocin induction and uncomplicated vaginal delivery	NR	NR	Gemfibrozil to treat hypertriglyceridemia
Gosseaume 2023 [27]	11 women (at least 1 pregnancy), median age: 25 years	FPLD3 (*PPARγ* gene mutation). Diabetes (median HbA1c 8.1%), hypertriglyceridemia (median TG 5.6 mmol/L), hypertension, pancreatitis, hepatic steatosis	Metabolic alterations, diabetes (n = 4), hypertension (n = 5), hypertriglyceridemia (n = 10), preeclampsia (n = 3)	6/25 pregnancies did not lead to birth. Premature delivery on average (<37 weeks), newborns’ birth weight was lower in maternal dysmetabolic environment (SGA vs LGA)	NR	Low leptin levels (median 8.4 ng/mL) before pregnancy	Most patients with diabetes are treated with insulin (56.5%)
Vantyghem et al 2008 [28]	14 women, range of age: 24-34 years	FPLD2 (Dunnigan variety-related to *LMNA* gene). 9/14 diabetes, 3/14 glucose intolerance, 8/14 hypertension, spaniomenorrhea, and hirsutism	Gestational diabetes and/or fetal macrosomia	7/14 at least 1 miscarriage; 3/14 at least 2 miscarriages; 2/14 eclampsia/fetal death. Mean number of live children per woman: 1.7	NR	Before pregnancy, low leptin levels (5.0 ± 3.8 ng/mL) in general. One case record: 18.2 ng/mL before and 16.9 ng/mL at 4 months of pregnancy. One case record: 9.9 ng/mL before and 15.7 ng/mL at 6 weeks of pregnancy. One case record: 3 ng/mL before and 4.7 ng/mL at 6 weeks of pregnancy	metformin and insulin in 4 case records
Valerio et al 2024 [29]	Eight women, range of age: 14-38 years	FPLD2 (Dunnigan variety-related to *LMNA* gene). 4/8 T2D, arterial hypertension, and PCOS. No fertility problems and extended efforts to conceive	2/8 gestational diabetes; 1/8 gestational hypothyroidism; 1/8 preeclampsia	Of 17 pregnancies (1 twin gestation), 2 miscarriages, 4 cases of premature birth; 15 live births, yielding to 16 newborns (1 set of twins); 5/16 LGA; neonatal complications included an unspecified malformation, respiratory infection; 4 cases of neonatal hypoglycemia; 2 neonatal deaths related to heart malformation and respiratory distress syndrome. Mean number of live children per woman: 1.75	Within a timeframe of 1-2 years following pregnancy, 3/4 patients were subsequently diagnosed with T2D, of whom 2 had a pregnancy with GD	NR	NR
Neal 2023 [30]	Seven women	One with FPLD2 (Dunnigan variety-related to *LMNA* gene); 1 with FPLD3 (*PPARγ* gene mutation); 1 with CGL1 (*AGPAT2* gene mutation); 4 with unknown genetic cause; 5/7 with pregestational diabetes	No acute pancreatitis. Gestational diabetes. Five cases of preeclampsia.	Of 21 pregnancies (19 singleton and 2 twin gestations), 10 successful pregnancies, 12 live births (7 to term, 3 preterm, 3 vaginal and 8 C-section deliveries), 11 miscarriages (9 first trimester, 2 second trimester in 1 patient due to cervical insufficiency) Of 12 live births, 1 SGA, 8 AGA, and 3 LGA; 6/12 neonatal hypoglycemia	NR	NR	NR

Abbreviations: AGA, appropriate for gestational age; CGL, congenital generalized lipodystrophy; C-section, cesarean section; FPLD, familial partial lipodystrophy; GD, gestational diabetes; GI, gastrointestinal; HbA1c, hemoglobin A1c; LGA, large for gestational age; NR, not reported; PCOS, polycystic ovary syndrome; SGA, small for gestational age; T2D, type 2 diabetes; TG, triglyceride.

Overall, the analysis included 48 women, aged 14 to 38 years, affected by various types of lipodystrophy: 27 cases of familial partial lipodystrophy type 2 (Dunnigan type related to *LMNA* gene) [21, 22, 25, 26, 28-30]; 15 cases of FPLD type 3 [peroxisome proliferator-activated receptors (*PPARγ*) mutation) [19, 24, 27, 30]; 4 cases of partial lipodystrophy of unknown genetic cause [30]; 3 cases of generalized lipodystrophy, of whom 1 was acquired [20] and 2 congenital (*AGPAT2* gene mutation) [23, 30]. Reported patients underwent a total of 78 pregnancies with a positive outcome. Of the 81 live newborns, some cases grew up in good condition after some initial complications [20, 23]. In 1 case, the child had developmental complications [24], and 11 cases reported neonatal hypoglycemia [29, 30]. Other problems, such as unspecified malformation, respiratory infection [29], and cases of neonatal death [19, 28, 29], were reported. The lipodystrophy disorder was usually accompanied by comorbidities, mainly including diabetes and hypertriglyceridemia, followed by arterial hypertension [19, 21, 28], pancreatitis [20, 24, 25], hepatic steatosis [19], preeclampsia [27, 29, 30], or abdominal discomfort [20]. Summarized cases reported some difficulties during pregnancy in managing pre-existing conditions, such as diabetes, hypertriglyceridemia, arterial hypertension, and pancreatitis. In 6 retrieved cases, new clinical conditions were developed during pregnancy [21, 24, 26, 27, 29]. The main clinical challenges during pregnancy concern achieving glycemic and TG control. In more detail, with the use of a high insulin dose and generally complex treatment, blood glucose levels could be maintained within a borderline diabetic range (HbA1c < 6%) [19-21, 23, 25]; however, poor or deteriorated glycemic control was also reported in some retrieved cases (HbA1c > 7.5%) [22, 24], whereas they have never been reported to improve as in our case. Concerning the management of hypertriglyceridemia, unlike our case where TGs increased less than expected, some retrieved cases have reported TGs to increase significantly at the onset of the pregnancy, leading to complications, such as pancreatitis: for instance, 20 to 100 mmol/L at week 6 and 12, respectively [24], or 1566 to 9975 mg/dL at week 7 and 12, lately ranged 1000 to 2000 mg/dL by treatment until birth [25].

Of the 79 pregnancies evaluated within these studies, it was possible to obtain information regarding the therapies applied during the pregnancy for only 37 cases. However, these data are still partial and fragmented. In the 23 cases (62.2%) resulting in live births, 18 (78.3%) required insulin therapy during pregnancy with variable doses (maximum 300 IU per day). Of all the pregnancies discussed, some reported the use, alone or in combination, of oral hypoglycemic therapy (n = 9, 24.3%; 9 metformin only, 1 metformin plus glibenclamide) [22]. To control dyslipidemia, the use of lipid-lowering drugs was reported in 5 cases (13.5%; 3 fenofibrate, 1 gemfibrozil) [26], 1 gemfibrozil shifted to fenofibrate [25], and 1 atorvastatin [24]. In only 1 case there was the need for the use of estradiol (0.5 mg, transdermal therapeutic system) and progesterone and subsequently of steroid (hydrocortisone and then prednisone) [20]; in another case, the use of an antihypertensive drug (nifedipine) due to persistent hypertension was reported [21]. The use of leptin therapy was also reported in only 5 cases (13.9%).

A premature delivery often occurred around week <37, also via cesarean section [20, 22, 24, 29, 30]. Except for 1 case [20] where postpartum complications caused the mother's death, clinical conditions generally improved and normalized after childbirth.

Only a few cases investigated the patient's leptin levels. Otherwise, in FPLD2 subjects, leptin values were monitored before or at an early stage of pregnancy; further values during the second or third trimester were not reported. One case showed exceptionally normal leptin values (18.2 ng/mL) before pregnancy, which were preserved at 4 months of pregnancy (16.9 ng/mL) [28]. Low leptin levels (3-9.9 ng/mL) were otherwise generally observed before [27, 28] or at an early stage of pregnancy [19], which increased during the first weeks of the gestational phase in 2 cases (9.9 and 3 ng/mL prepregnancy, 15.7 and 4.7 ng/mL at 6 weeks of pregnancy, respectively) [28].

## Discussion

Lipodystrophy disorder is classified as genetic or acquired, partial or generalized, whose manifestations are typically accompanied by comorbidities, including metabolic impairment, insulin resistance, and hypertriglyceridemia [1]. In this heterogeneous scenario, pregnancy represents an additional challenge in managing such a complex condition. We described a complex case of APL secondary to BMT with an unprogrammed pregnancy and compared it with the published literature.

First, our case aligned with the literature, resulting in a live female newborn. Most of the described patients, including our case, had successful pregnancies, although the clinical and metabolic pictures were frequently complicated at the start and during pregnancy. Case reports indicated a high risk for people with lipodystrophy subtypes, including several types of genetic partial lipodystrophies [19, 21, 22, 24, 25, 27, 28, 30], congenital generalized lipodystrophies [23, 30], Köbberling syndrome [26], and another case of acquired generalized lipodystrophy [20]. Therefore, pregnancy is a debatable option for these patients. Miscarriage or death of a child after delivery is a meaningful event (31 events, prevalence of 26.3% on total pregnancies) [20, 22, 27, 28, 30] that should be taken into account and evaluated for the impact on quality of life.

Second, most of the cases, including our patient, experienced a preterm emergency delivery ranging from 28 to 37 weeks of gestation [19, 20, 22-24, 27, 29, 30], and many babies had partum or postpartum transient or chronic complications, including neurological injuries, or had chromosome disorders, as in our case. These findings suggest that follow-up should be organized with many specialist practitioners when a pregnancy is planned. Experienced emergency gynecologists and pediatricians should collaborate closely with physicians who follow young females with lipodystrophy during their reproductive phase.

According to the results of our literature search, except for 3 cases of generalized lipodystrophy [20, 23, 30], we retrieved cases concerned FPLD2 and FPLD3 related to *LMNA* or *PPARγ* mutation, respectively. These findings could suggest that pregnancy research could be more successful in partial lipodystrophies than in generalized cases. However, infertility problems, oligo/amenorrhea, and polycystic ovary syndrome are frequently reported [28-32]. On the other hand, unreported bias could not be excluded. Furthermore, our case is the first to date to report on a patient with APL following allogenic BMT during her pediatric age. Other retrieved cases concerned acquired (general) forms of lipodystrophy with unknown cause (likely autoimmune) [20].

Different genetic mutations can be correlated with specific forms of lipodystrophy, phenotypic, and clinical features, as well as diverse severity grades of the disease [33-35], and these characteristics seem to be maintained even during pregnancy. Fetal/neonatal death or nonmetabolic complications may be related to a fetal dysmetabolic environment of maternal origin over the pregnancy (see our case) or to the specific genes involved in the phenotype. In particular, a functional PPAR*γ* protein is reported to be essential for placental angiogenesis and fetal growth [36]. In 1 retrieved case reporting on a neonatal death, the *PPARγ* mutation inherited from placental tissues was responsible for the abnormal placentation and the fatal outcome, suggesting that FPLD3 pregnancies may be at high risk, especially if the fetus has inherited the mutation [19, 37-40]. The causes of an increased risk of fetal/neonatal mortality and morbidity or birth weight, as observed in retrieved cases, have not been deeply investigated so far. Several pathogenic mechanisms could be involved, including effects of specific genes, metabolic dysfunctions, inflammation, epigenetic alterations, inadequate adaptation of the organism to the pregnancy, and altered homeostasis between the dyad unable to sustain energy delivery for fetus development and growth.

Metabolic dysfunctions are always present in the described population, starting from diabetes and hypertriglyceridemia and followed by arterial hypertension [19, 21, 28-30], pancreatitis [20, 25], hepatic steatosis [19], or abdominal discomfort [20]. Furthermore, the metabolic phenotype of retrieved cases and thus their etiopathogenesis vary before and during pregnancy, also suggesting other potential issues than lipodystrophy playing a role in pregnancy management and outcome. The main clinical challenges during pregnancy concerned achieving glycemic and TG control, whereas in our case, glucose levels progressively improved, and TGs increased less than expected. In some cases [23, 24, 26, 27, 29, 30], new conditions have developed. The complexity of the treatments varies among cases. However, a few details were present on the type of drugs in several of the papers.

Regarding *PPARγ* mutations, women carried out 1 or several pregnancies, all complicated by diabetes, hypertension, and/or hypertriglyceridemia [24, 27, 30] and frequently reported small for gestational age babies, when a fetal exposition to a dysmetabolic environment of maternal origin was present. This may suggest the potential role of lipodystrophy-related *PPARγ* variants in inducing early metabolic complications. These mothers probably require supraphysiological insulin doses to maintain glycemic control and prevent hypertriglyceridemia and resultant pancreatitis; this titration strategy could impact the final baby weight or polyhydramnios presence.

Women affected by Dunnigan syndrome also carried out 1 or several pregnancies in the presence of clinical complications, such as diabetes, hypertension, hypertriglyceridemia, and/or proteinuria, the latter not reported in pregnancy in patients affected by FPLD3 [21, 22, 25, 28-30]. Large babies were more prevalent than appropriate for gestational age or small for gestational age, suggesting that mechanisms impairing fetal growth could be partially different from those with FPLD3. Several women achieved pregnancy via treatment with insulin sensitizers and less need for insulin, probably linked to a specific insulin-resistant phenotype, as also demonstrated by the prevalence of polycystic ovary syndrome in these patients [28, 29]. Metabolic conditions can change during pregnancy: in the first half, higher insulin sensitivity coupled with an anabolic state and increased lipid storage are observed, whereas, during the second half, insulin resistance starts, resulting in a catabolic state that favors energy from glucose, amino acids, and lipids for the fetus. In a healthy pregnancy, this insulin resistance is usually balanced by pancreatic positive feedback, leading to a high rate of insulin secretion due to β-cell hyperplasia [41]. Therefore, it has been hypothesized that estrogens could be a master regulator of insulin-resistance grade in *LMNA* mutated patients during pregnancy since the phenotype typically starts from puberty and is more aggressive in females than males [1, 28, 42].

Here we report the first described case of pregnancy in a patient affected by APL secondary to an allogenic BMT that occurred at pediatric age. BMT represents a high-risk factor for APL due to several reasons linked to the cancer history, including total body irradiation, graft vs host disease, glucocorticoid treatment, CT, and stem cell transplant. All these events could contribute to the inhibition of preadipocyte expansion, fat loss at the subcutaneous adipose tissue level, and preferential migration of the mesenchymal stem cells transplanted to the visceral adipose tissue [16, 43]. Some of the patients with this form of APL present a Cushing phenotype, suggesting that glucocorticoid treatment may have contributed partly to the phenotype—mainly alterations in body distribution since weight gain or obesity is not reported in the literature in these cases [43]. Besides adipose tissue dysfunction, these patients could develop many metabolic complications and cardiovascular diseases secondary to cancer treatments [44]. Our patient suffered from an acute pulmonary edema and required an emergency preterm delivery via cesarean section at 33 weeks. The event was coupled with a severe left ventricular diastolic dysfunction (EF 30%) with left ventricular dilatation and depression of systolic function that was treated invasively. Since the cardiac magnetic resonance imaging failed to detect alterations in tissue characterization or adipose tissue depots, cardiac injury was a probable consequence of previous treatment with anthracyclines, suggesting how managing these patients is a multidisciplinary challenge. Transient hypopituitarism was also observed without starting lactation, probably due to the acute hemodynamic alterations [45].

First, at the baseline visit in our clinic, the patient had suffered from a long history of amenorrhea with maintained gonadotropin secretion but low estrogen levels, likely due to a partial leptin insufficiency or leptin asynchrony, since it has been demonstrated that leptin regulates LH and estradiol oscillations [46]. The quick restoration of ciclicity after the partial improvement of glucose control was quite unexpected. We cannot exclude a role of the introduction of semaglutide in her daily treatment. There is evidence that glucagon-like peptide (GLP)-1 inhibits appetite in part through regulation of soluble leptin receptors and that it can restore leptin responsiveness [47]. Furthermore, several studies have demonstrated that GLP-1 and its analogs, beyond improving body weight and metabolic health, regularize menstrual cycling and improve pregnancy rates and outcomes, acting both on gonadotrophins and ovary [48].

Before pregnancy, the patient presented hypertriglyceridemia and lipodystrophy-related diabetes that were not controlled, although she was on therapy. Unexpectedly, glucose levels progressively improved during the pregnancy, and TGs increased less than expected, which are 2 of the main clinical conditions associated with lipodystrophy representing a challenge for clinical care, as discussed before. To explain this phenomenon, we started to measure leptin levels. Interestingly, increasing levels were observed, in our case, with a peak in the third trimester (41.53 ng/mL), followed by a fast decline the day after giving birth, with a lower basal level than the prepregnancy period. If a few reports described pregnancy in patients with lipodystrophy, a minority of them reported leptin levels during pregnancy. A paucity of cases in FPLD3 and FPLD2 reported leptin values only in the early stage of the first trimester. Similarly to our case (acquired form), these retrieved cases of congenital forms reported initially a stable or slight increase of leptin levels; however, they did not investigate them afterward [19, 28]. Indeed, we have no comparisons to discuss in depth the increase in leptin levels that we recorded in the first and second trimesters of gestation in our case. Since the hormone abruptly dropped down after the delivery, we hypothesized that the increase in the blood was due to placental and/or fetal origins. Leptin and its receptor are both expressed in the placenta, modulating invasions, nutrient transport to the fetus, and healthy fetal growth. Abnormal high levels of expression are associated with the risk of cesarean section, preterm birth, and preeclampsia [49, 50]. Too high leptin levels are also associated with gestational diabetes and macrosomia [51].

We speculated that placental production of leptin in our patient balanced the partial deficiency due to subcutaneous fat loss observed before and after pregnancy, contributing to her metabolic improvement despite discontinuing several drugs, such as semaglutide and fibrate, for safety reasons. We cannot exclude that the same phenomenon contributes to the phenotype in pregnancy, at least in patients with lipodystrophy due to *LMNA* mutations, although tailored studies are needed. Although this is a hypothesis, it is supported by several physiological findings. During pregnancy, the mother preferentially uses fats and fat storage for energy while conserving the fetus's access to glucose and amino acids. Consequently, lipolysis is increased, and TGs are used to meet her energy requirements. It is possible that, in our case, coupled also with the increase in leptin, insulin resistance did not overcome the physiological threshold, and the change in metabolic fuels related to the placenta favored the decrease in TGs we observed. In parallel, the increased peripheral use of glucose by the fetus, the increased glycogen storage, and the decreased glucose production by the liver could have contributed to the improvement in glucose metabolism. Different expressions of glucose transporters and insulin receptors in the placenta could contribute to the phenomenon [52, 53]. Furthermore, the treatment with GLP-1 analog could be hypothesized to have a role in the increase of leptin during pregnancy in our patient, since it modulates leptin circulating levels, as discussed before [47]. All of these speculations deserve further study.

We cannot easily compare the clinical phenotype of our patients with respect to genetic forms and other acquired forms of lipodystrophy since, regarding the latter etiology, only another case has been published [20]. However, also in this case, severe hypertriglyceridemia and diabetes were present before pregnancy with a following acceptable metabolic trend during the gestation, although an urgent cesarean section at 28 weeks was needed. Metabolic features deteriorated 6 months after the delivery such as in our case. The primary pathogenesis of lipodystrophy may influence the metabolic and organic features during pregnancy. Information derived from wide cohorts and registries is warranted to explore these aspects.

This work has some limitations. Only cases described in the last 25 years were included, and several other studies had been previously published. However, very old cases are classified too frequently only as lipoatrophic diabetes, reducing the possibility of phenotyping them [54-56]. Moreover, in our case, leptin values were not measured in the cord blood and/or placental expression during delivery due to the emergency cesarean section and the mother's compromised hemodynamic conditions. On the other hand, our study was the first one to describe leptin levels during the last months of pregnancy in an APL.

In conclusion, despite the positive outcome and eventual clinical recovery and improvement or stability in most cases, pregnancy in the presence of a lipodystrophy disorder exposes both the mother and the child to very high risks. Therefore, achieving prepregnancy metabolic control and a meticulous care plan for the intense multispecialty monitoring of the subject before, during, and after pregnancy is required. The regulation of leptin secretion during pregnancy and its potential therapeutic modulation in different phenotypes of lipodystrophy should be further investigated.

## Data Availability

Original data generated and analyzed during this study are included in this published article or in the data repositories listed in References. Further inquiries can be directed to the corresponding author.

## Abbreviations

AGP: ambulatory glucose profile
APL: acquired partial lipodystrophy
BMT: bone marrow transplant
CT: chemotherapy
EF: ejection fraction
FPLD: familial partial lipodystrophy
GLP: glucagon-like peptide
GMI: glucose management indicator
HbA1c: hemoglobin A1c
IT: intrathecal
MTX: methotrexate
PPAR: peroxisome proliferator-activated receptors
TAR: time above range
TBR: time below range
TG: triglyceride
TIR: time in range
